# Formylpeptide Receptors Mediate Rapid Neutrophil Mobilization to Accelerate Wound Healing

**DOI:** 10.1371/journal.pone.0090613

**Published:** 2014-03-06

**Authors:** Mingyong Liu, Keqiang Chen, Teizo Yoshimura, Ying Liu, Wanghua Gong, Yingying Le, Ji-Liang Gao, Jianhua Zhao, Ji Ming Wang, Aimin Wang

**Affiliations:** 1 Department of Spine Surgery, Daping Hospital, Third Military Medical University, Chongqing, China; 2 Laboratory of Molecular Immunoregulation, Cancer and Inflammation Program, Center for Cancer Research, National Cancer Institute, Frederick, Maryland, United States of America; 3 National Center For Nanoscience and Technology, Beijing, China; 4 Basic Research Program, Science Applications International Corporation-Frederick, Frederick, Maryland, United States of America; 5 Key Laboratory of Food Safety Research, Institute for Nutritional Sciences, Shanghai Institutes for Biological Sciences, Chinese Academy of Sciences, Shanghai, China; 6 Laboratory of Molecular Immunology, National Institute of Allergy and Infectious Diseases, Bethesda, Maryland, United States of America; 7 The Center for Orthopedics, Daping Hospital, Third Military Medical University, Chongqing, China; French National Centre for Scientific Research, France

## Abstract

Wound healing is a multi-phased pathophysiological process requiring chemoattractant receptor-dependent accumulation of myeloid cells in the lesion. Two G protein-coupled formylpeptide receptors Fpr1 and Fpr2 mediate rapid neutrophil infiltration in the liver of *Listeria*-infected mice by sensing pathogen-derived chemotactic ligands. These receptors also recognize host-derived chemotactic peptides in inflammation and injury. Here we report the capacity of Fprs to promote the healing of sterile skin wound in mice by initiating neutrophil infiltration. We found that in normal miceneutrophils rapidly infiltrated the dermis in the wound before the production of neutrophil-specific chemokines by the injured tissue. In contrast, rapid neutrophil infiltration was markedly reduced with delayed wound closure in mice deficient in both Fprs. In addition, we detected Fpr ligand activity that chemoattracted neutrophils into the wound tissue. Our study thus demonstrates that Fprs are critical for normal healing of the sterile skin wound by mediating the first wave of neutrophil infiltration.

## Introduction

Wound healing is an interactive process that involves soluble mediators, components of extracellular matrix, resident cells (epithelial cells, fibroblasts, endothelial cells, nerve cells), and infiltrating leukocyte subsets. Wound healing is composed of three phases: inflammation, tissue formation, and tissue remodeling [1]. Inflammatory reaction during wound healing does not subside with epithelialization, but rather, persists until tissue remodeling, with a different cellular composition as opposed to the early acute phase. Skin is a standard site of wound healing model, in which the leukocyte subsets, as the cellular components of inflammation, are not only immunological effector cells against invading environmental bacteria but are also involved in the anabolic phase of tissue degradation through the production of proteases and reactive oxygen intermediates and, in particular, in the catabolic phase of tissue formation through production of growth factors. Therefore, the recruitment of leukocytes is critical during the inflammatory reaction in wound healing process.

Tissue injury is associated with rapid accumulation of neutrophils which constitute nearly 50% of all cells at the wound site [2]. The accumulation of neutrophils is controlled by multiple chemoattractants [2], [3], such as IL-8 (CXCL8), neutrophil-activating peptide-2 (NAP-2;CXCL7) [4], and growth-related oncogene α (GRO-α) (CXCL1) [5], [6], which use G protein-coupled chemokine receptors (GPCRs) expressed by neutrophils. Neutrophils also express other GPCRs, namely formylated peptide receptors (FPRs) that recognize a plethora of pathogen- and host-derived chemotactic and activatingmolecules [7], [8]. Neutrophils express two FPRs, FPR1 and FPR2 in human and Fpr1 and Fpr2 in mice. Activation of FPRs or Fprs by their agonists elicits a signaling cascade that culminates in neutrophil migration, increased phagocytosis and release of superoxide. In mouse disease models, Fprs have recently been implicated in sustaining innate and adaptive immune responses, promoting host defense against bacterial infection by mediating rapid infiltration of neutrophils, maintaining the homeostasis of colon epithelia, and polarizing macrophages toward M1 phenotype in a malignant tumor [8], [9], [10], [11]. Therefore, FPRs (Fprs) actively participate in diverse pathophysiological processes. However, whether FPRs (Fprs) accelerate wound closure by recruiting neutrophils into the wound is unknown. In this study, we examined the role of Fpr1 and Fpr2 in the healing of full-thickness skin wound in mice by using mouse strains deficient in Fprs. Here we report that Fpr1 and Fpr2 cooperatively participate in skin wound healing by mediating the first wave of neutrophil accumulation.

## Materials and Methods

### Animals

Mouse strains deficient in Fpr1 (Fpr1^-/-^), Fpr2 (Fpr2^-/-^) or both Fpr1 and Fpr2 (Fpr1/2^-/-^) were generated as described [9], [12] (Fpr1 mice were kindly provided by Dr. P. Murphy of NIAID, NIH, USA). Mice were backcrossed to C57/Bl6 background for at least 8 generations before use. All mice were housed in the animal facility at National Cancer Institute and were used at 8–12 week of age.

This study was carried out in strict accordance with the recommendations in the Guide for the Care and Use of Laboratory Animals of the National Institutes of Health, USA. Mouse experiments were approved by Animal Care and Use Committee of National Cancer Institute and performed in accordance with the procedures outlined in the “Guide for Care and Use of Laboratory Animals” (National Research Council; 1996; National Academy Press, Washington D.C.). All surgery was performed under ketamine and xylazine anesthesia, and all efforts were made to minimize suffering.

### Wound repair model

The wound repair model was previously described [13], [14], [15]. Mice were anesthetized by intra-peritoneal (IP) injection of ketamine (Sigma-Aldrich, 80 mg/kg) plus xylazine (Sigma-Aldrich, 10 mg/kg) [13].The skin on the mouse back was cleaned, shaved, and sterilized with betadine solution followed by 70% ethanol. A 6-mm full-thickness (including the Panniluluscarnosus) excisional punch biopsy (AcuPunch, Fort Lauderdale, FL) was performed on the right and left upper paravertebral regions of each animal [15]. The biopsy sites were covered with non-adhesive sterile gauze. Mice were wrapped with a form-fitting bandage to further protect the biopsy sites. In a series of experiments, mice were injected IP with an Fpr2 agonist CRAMP (Cathelicidin antimicrobial peptide, 10 µg/100 µl) or a pan Fpr antagonist Boc-2 (5 µM, 100 µl) daily starting 3 days before biopsy. Wounds were examined daily for infection and photographed using a Nikon 9000D digital camera (Nikon, Japan). Changes in wound contraction over time were calculated using an NIH Image J software (version 1.37). Results are obtained from a minimum of six independent animals/group.

### Immunofluorescence

Neutrophils were identified by immunofluorescence using a rat anti-mouse Ly6G Ab (eBioscience) followed by AlexaFluora 488-labelled goat anti-rat IgG. Nuclei were stained with 4`,6-diamidino-2-phenylindole (DAPI, Invitrogen). Goat IgG (eBioscience) was used as an isotype control.

### Chemokine production

Skin specimens (1 cm×1 cm) with injured site in the center were cut and homogenized in 5 ml DPBS. The homogenates were centrifuged at 12000 rpm for 5 min and the supernatants were collected for measurement of neutrophil chemokines CXCL1 and CXCL2 by ELISA (R&D) or for chemotactic activity at different dilutions.

### Isolation of neutrophils

Mice were euthanized and bone marrow was harvested by flushing the femurs and tibias with DPBS.Bone marrow cells were cultured in RMPI 1640 supplemented with 2 mM l-glutamine, 50 mg/ml gentamicin,fetal bovine serum (10%) and GM-CSF (20 ng/ml) after tissue debris was filtered out and red blood cells were lysed with ACK lysing buffer. Culture medium was replaced each other day and cells were harvested for chemotaxis assay on day 6. The purity of neutrophils was >90%.

### Chemotaxis

The chemotaxis of neutrophils and HEK293 cells transfected with Fprs was analyzed using polycarbonate membranes with 3-μm (neutrophils) or 8-μm pore size (HEK293 cells) in 48-well chambers (NeuroProbe, Gaithersburg, MD). An aliquot of 29 μlchemoattractants was placed in the lower wells of the chamber, and 50 µl of cells (1.5×10^6^/ml) suspended in RPMI 1640 with 0.5% BSA were placed in the upper wells. After incubation (45 min for neutrophils and 240 min for HEK293 cells) at 37°C, membranes were removed, rinsed with PBS, fixed, and stained with Diff-Quik 3-step stain solution. Migrated cells were counted in 3 random fields at 400 magnification under light microscopy. Neutrophils from WT mice were also pretreated with Fpr antagonists (Boc-1 for Fpr1, WRW4 for Fpr2 and Boc-2 for Fpr1 and Fpr2) at different concentrations then measured for chemotaxis in response to chemokines and skin homogenate supernatants.

### Statistical analysis

All experiments were repeated at least three times with reproducible results. Results shown were from representative experiments. Statistical differences between testing and control groups were analysed by Student's *t*-test. A P value < 0.05 was considered as statistically significant.

## Results and discussion

### Fpr-deficiency delays skin wound healing

**Figure 1:**
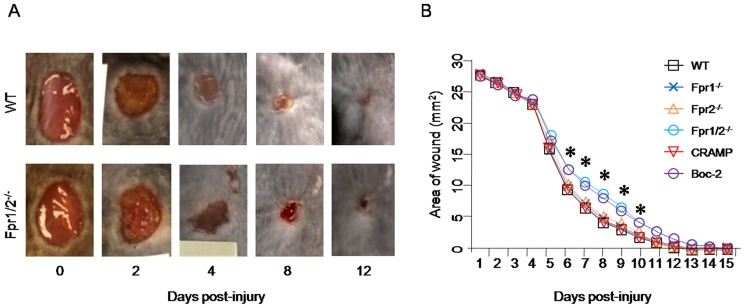
Delayed skin wound healing in Fpr double deficient Fpr1/2^-/-^mice. *A*, representative pictures of skin wound of WT and Fpr1/2^-/-^ mice. A 6-mm full-thickness (including the Panniluluscarnosus) skin was excised from the right and left upper paravertebral regions of each animal and the injured areas were measured daily using NIH Image J software (version 1.37) (n = 5). *B*, the areas of wounds. Mice were punched to generate two 6-mm full thickness skin wounds with or without IP injection CRAMP (10 µg/100 µl) or Boc-2 (5 µM, 100 µl) (n = 15). The areas of wound were calculated. *, significantly increased wound area in Fpr1/2^-/-^and Boc-2 treated mice, compared with the wound area of WT mice at the same time points (*p*< 0.05).

We first examined the natural healing process of skin wound in WT mice. The wound contraction started from day 1 after injury and healing accelerated from day 4 through day 10. The wound was completely healed within 12 days in WT mice (**Fig. 1A**). In contrast, the healing of skin wound in Fpr1/2^-/-^mice was significantly delayed as compared with WT mice. The areas of the wound in Fpr1/2^-/-^ mice were significantly larger than in WT mice from day 6 until day 10 (**Fig. 1B**). However, in Fpr1 or Fpr2 single deficient mice, no significant differences in the healing were observed as compared with WT mice, suggesting that lack of a single Fpr was not sufficient to cause the delay in normal skin wound healing. Injection of an Fpr2 agonist, CRAMP, in WT mice did not accelerate the healing. However, a pan-Fpr antagonist, Boc-2, significantly delayed the healing of the skin wound in WT mice (**Fig. 1B**). These results indicate that normal skin wound healing requires the participation of both Fpr1 and Fpr2.

### Fpr double-deficiency impairs neutrophil infiltrationin skin wound

**Figure 2:**
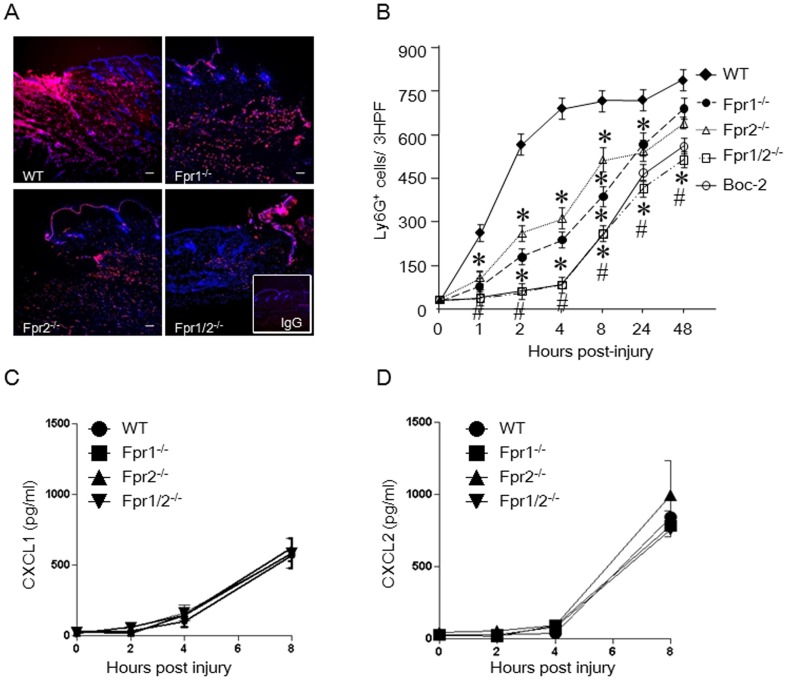
Reduced neutrophil infiltration in the wounds of Fpr-deficient mice and the production of chemokines. *A*, representative immunofluorescence of skin wound showing Ly6G^+^ cells 4 h after injury. Cryosections of wounded skin from WT and Fpr-deficient mice were labeled with Ly6G and DAPI (Red: Ly6G; Blue: DAPI) (n = 5, scale bar: 20 µm). Insert: control IgG staining. *B*, Kinetics of infiltrating Ly6G^+^ neutrophils in 3 consecutive high power fields (HPF). *, significantly reduced Ly6G^+^ cells in the wounds of Fpr-deficient micecompared with WT mice (*p*< 0.05). #, significantly reduced Ly6G^+^ cells in the wounds of Boc-2 pretreated WT mice, compared with WT mice without pretreatment (*p*< 0.05). *C&D*, chemokinesCXCL1 and CXCL2 in the homogenates of skin wound from WT and Fpr-deficient mice. Skin (1 cm×1 cm) with wounds in the center was excised and homogenized in 5 ml DPBS. The homogenates were centrifuged and the supernatants were collected for measurement of CXCL1 and CXCL2 by ELISA (n = 15).

In investigating the mechanisms involved in Fpr-promoted wound healing, we detected a rapid wave of neutrophil accumulation in the WT mouse skin wound, beginning at 60 min and peaking at 4 h post injury (**Fig. 2A**). In contrast, in the wounds of Fpr double-deficient (Fpr1/2^-/-^) mice, neutrophil accumulation was markedly reduced. Despite subsequent slow increase of neutrophils in the injured site of Fpr1/2^-/-^ mice up to 48 h, the cell number remained significantly lower than in WT mice (**Fig. 2B**). Injection of a pan-Fpr antagonist, Boc-2, completely abrogated the rapid neutrophil infiltration in WT mouse wound, suggesting the production of Fpr agonist(s) in the wound (**Fig. 2B**). It is interesting to note that there was also a delay in the first wave neutrophil infiltration in the wound of Fpr1^-/-^ or Fpr2^-/-^ mice. However, this delay was not as severe as in Fpr1/2^-/-^ mice and did not affect the progress of wound healing in Fpr single deficient mice, as shown in the kinetics of skin wound closure.

**Figure 3:**
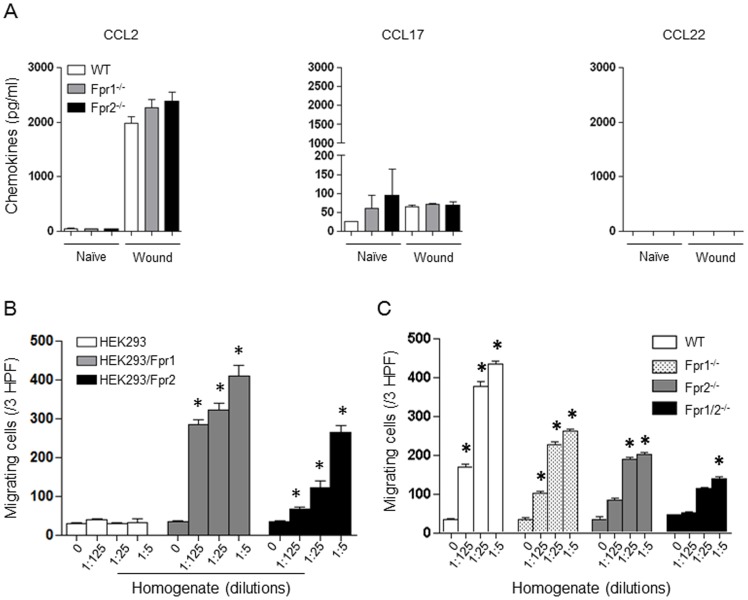
Chemokine production and chemotactic activity of homogenates of skin wound. *A-C*, chemokine production of skin wound at 72 h (n = 15). WT and Fpr-deficient mice were subjected to full-thickness skin wound and the wounds were harvested at 72 h after injury and then homogenized for chemokine measurement with ELISA. *D*, homogenate-induced migration of parental and Fpr-transfected HEK293 cells. Migrating cells in response to different concentration of the homogenate in 3 HPF were counted. *, significantly increased migrating cells in response to homogenates as compared to medium control (0) (*p*<0.05). *E*, chemotactic activity of skin homogenates for neutrophils from WT and Fpr-deficient mice. *, significantly increased migrating neutrophils in response to homogenates as compared to medium control (0) (*p*<0.05).

We then examined the nature of chemoattractants responsible for neutrophil infiltration in the wounds. Since chemokine GPCRs are demonstrated to play a major role in neutrophil infiltration in inflammatory responses [16], [17], [18], [19], [20], [21], [22], [23], we measured the production of neutrophil specific chemokines, CXCL1 and CXCL2, in the homogenates of injured skin. In the lesion of WT mice, despite a rapid infiltration of neutrophils, the production of CXCL1 and CXCL2 was not detectable at 4 h post injury and there was no difference in CXCL1 and CXCL2 levels in the wounds of Fpr-deficient mice and WT mice until 72 h post injury (**Fig. 2C&D** **, and Figure S1**). Other chemokines such as CCL2, CCL17, but not CCL22, were also detected but no significant differences were found between WT and Fpr-deficient mice (**Fig. 3A-C** **, and Figure S2**). These results indicate that the “first wave” neutrophil infiltration in the skin wound of WT mice is not dependent on chemokines, but rather, Fpr ligands are likely responsible.

### Fprs are major receptors on neutrophils to sense chemotactic signals in early skin wound

**Figure 4:**
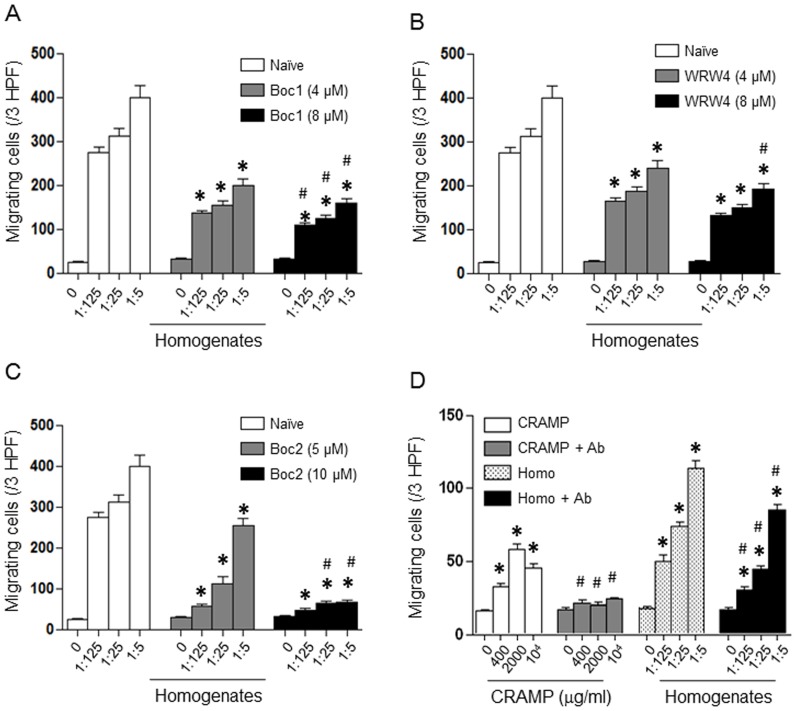
Fpr agonist activity in the skin wound. *A - C,* inhibition of homogenate-induced migration of neutrophils from WT mice by Fpr antagonists (n = 5). Neutrophils from WT mice were pretreated with Boc-1 (4 µM and 8 µM, *A*), WRW4 (4 µM and 8 µM, *B*) or Boc-2 (5 µM and 10 µM, *C*) for 30 min and then were measured for chemotaxis in response to homogenates of wounded skin collected at 4 h. *, significantly decreased number of migrating cells treated with Fpr antagonists as compared to neutrophils without pretreatment (*p*< 0.05). #, significantly decreased number of migrating cells treated with Fpr antagonists as compared to neutrophils without pretreatment (*p*< 0.05). *D*, inhibition of the chemotactic activity of skin wound homogenates by a neutralizing CRAMP antibody. HEK293/Fpr2 cells were measured for chemotaxis in response to CRAMP or skin wound homogenates with or without pretreatment by a neutralizing CRAMP antibody (10 µg/ml for 45 min) (n = 5). *, significantly increased number of migrating cells in response to CRAMP or homogenates as compared to medium control (0) (*p*< 0.05). #, significantly decreased number of migrating cells as compared to neutrophils without pretreatment by CRAMP antibody (*p*< 0.05).

To examine the presence of FPR ligands in the skin wounds, we measured Fpr agonist activity produced in the lesion. The homogenates of mouse skin4 h after punch, when there was little CXCL1 and CXCL2 production, induced marked migration of HEK293 cells transfected to express Fprs, but not the parental HEK293 cells (**Fig. 3D**), indicating that homogenates contain ligands for both Fpr1 and Fpr2. WT mouse neutrophils also migrated potently in response to the skin homogenates (**Fig. 3E**). In contrast, Fpr1^-/-^ or Fpr2^-/-^ mouse neutrophils each showed reduced chemotaxis to skin homogenates, with greater reduction of the migration of Fpr1/2^-/-^ mouse cells. All Fpr-deficient mouse neutrophils retained normal chemotaxis in response to ligands using other GPCRs [8], including CXCL1 and CXCL2. These observations corroborate the notion indicating the production of Fpr1 and Fpr2 agonists at the sites of injured skin. The observation that neutrophils from Fpr1/2^-/-^ mice also showed low level chemotactic response to skin homogenates suggests that skin wound homogenates may contain chemoattractants using receptors other than Fpr1 and Fpr2, such as chemokines produced 4 h after wounding. To clarify the nature of Fpr agonists in the skin homogenates, neutrophils from WT mice were pre-treated with Fpr specific antagonists (Boc-1 for Fpr1, WRW4 for Fpr2 and Boc-2 for both Fpr1 and Fpr2) before measurement of chemotaxis in response to the homogenates. The results showed that Boc-1 (**Fig. 4A**) and WRW4 (**Fig. 4B**) each partially inhibited homogenate-induced neutrophil migration. The pan-Fpr antagonist, Boc-2, showed greater inhibition of neutrophil migration in response to the homogenates of injured skin (**Fig. 4C**). Thus, Fpr1 and Fpr2 specific agonists are released in the lesion of mouse skin, which are likely responsible for the rapid infiltration of neutrophils. It is interesting to note that neutrophils also show chemotactic responses to skin homogenates even in the presence of high concentrations of the pan Fpr antagonist Boc-2, indicating the involvement of other chemoattractant receptor(s) expressed by neutrophils. This is consistent with the results showing remaining low level chemotactic response of Fpr1/2^-/-^ neutrophils to the skin homogenate (**Fig. 3C**). To further clarify the nature of the chemoattractants in skin homogenates, a neutralizing antibody against the Fpr2 agonist CRAMP was used that completely abrogated the capacity of CRAMP to induce the migration of HEK293/Fpr2 cells, and substantially inhibited the chemotactic activity of skin homogenate supernatant for HEK293/Fpr2 cells and neutrophils (**Fig. 4D**). Experiments with neutrophils yielded similar results in terms of antibody neutralization of the agonist activity in the skin homogenates (data not shown). Thus, CRAMP is involved in the induction of infiltration of neutrophils in skin wound. The fact that anti-CRAMP only partially inhibited the chemotactic activity of the skin homogenates for Fpr2 transfected 293 cells suggests that Fpr2 ligand(s) in addition to CRAMP may exist. Further effort is being made to characterize the molecular nature of such unknown agonists for Fpr2. Also, the nature of potential Fpr1 agonist(s) in the wound tissue remains to be determined.

In our study, although Fpr1 and Fpr2 single deficiency did not show significant impact on the rate of wound closure in mouse skin, lacking both Fprs resulted in a more severe reduction in early neutrophil infiltration and significant delay in the healing of the skin wound. These results suggest that a normal wound healing process requires the participation of both Fpr1 and Fpr2 for early neutrophil recruitment and subsequent wound closure.Consistent with our findings, Fpr1 was reported to mediate rapid neutrophil accumulation in patients with trauma-induced systemic inflammatory response syndrome [24]. In fact, it has been reported that multiple chemoattractants regulate neutrophil trafficking in pathophysiological conditions. Platelets entrapped and aggregated in the blood clot release, among growth factors such as platelet-derived growth factor (PDGF), the chemokine-connective tissue-activating peptide-III (CTAP-III), which is converted proteolytically into neutrophil-activating peptide-2 (NAP-2;CXCL7) by neutrophils attached to the thrombus [4], acts as a first-line mediator of neutrophil recruitment via the CXC chemokine receptor 2 (CXCR2) [4]. In the present study, Fpr1/2 deficient mice showed markedly delayed wound healing with normal CXCL7 responsiveness, suggesting that CXCL7 is not responsible for the rapid early neutrophil accumulation in the skin wound. In addition, secretion of CXCL1 (growth-related oncogene α, GRO-α) [5] and CXCL5 [3] have also been reported to support neutrophil recruitment in the late phase of wound healing.Furthermore, Toll-like receptors (TLRs), TLR3 and 7, which sense viral double- and single-stranded RNA, promote neutrophil infiltration in the wound by stimulating chemokine production, but in a much later phase after injury [25]. These observations support the importance of our findings of Fprs as the sensors of tissue-derived agonists for rapid neutrophil accumulation in the wound. Recently, Fpr1has been shown to complete a chemotaxis signal relay with other GPCRs to guide neutrophil infiltration in sterile liver injury [26]. In the lung of allergic inflammation, chemokine receptor CCR2 elicits initial accumulation of monocyte-derived dendritic cells (DCs) in peri-vascular region, but Fpr2 is critical for cell trafficking to peri-bronchiolar area where mature DCs gain markedly enhanced function of CCR7 for final trafficking into draining lymph nodes [27]. In a laser-induced sterile skin wound model, two-photon intravital microscopy showed that dead cells release factors, including LTB4, causing rapid neutrophil infiltration in 15 min followed by amplified neutrophil swarming in response to Fpr2 and CXCR2 ligands in the wounds [28]. Taken together, Fprs are of critical importance in many pathophysiological processes that involve neutrophil trafficking and may constitute molecular targets for therapies.

## Supporting Information

Figure S1 **Chemokine production in skin wound at 72 h.** WT and Fpr-deficient mice were subjected to full-thickness skin wound and the wounded skin area were harvested at 72 h after injury and then homogenized for chemokine measurement with ELISA.
(TIF)

Figure S2 **CCL2 production in skin wound.** WT and Fpr-deficient mice were subjected to full-thickness skin wound and the wounded skin areas were harvested and homogenized for CCL2 measurement with ELISA.
(TIF)
