# Correction: Formylpeptide Receptors Mediate Rapid Neutrophil Mobilization to Accelerate Wound Healing

**DOI:** 10.1371/journal.pone.0099541

**Published:** 2014-06-03

**Authors:** The PLOS ONE Staff

There is an error in the corresponding author designation. The correct corresponding authors are: Aimin Wang (trauma2@163.com), Ji Ming Wang (wangji@mail.nih.gov), and Jianhua Zhao (zhaojianhua194@yahoo.com).
