# Supplementary figures and images for: Formylpeptide Receptors Mediate Rapid Neutrophil Mobilization to Accelerate Wound Healing
Source: PLoS One. 2014 Mar 6;9(3):e90613. doi: 10.1371/journal.pone.0090613 (PMC3946181; doi:10.1371/journal.pone.0090613)

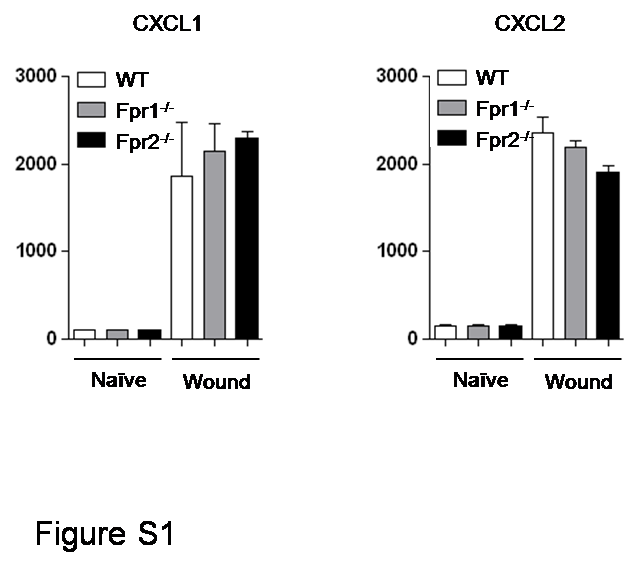

Supplement: Figure S1 — Chemokine production in skin wound at 72 h. WT and Fpr-deficient mice were subjected to full-thickness skin wound and the wounded skin area were harvested at 72 h after injury and then homogenized for chemokine measurement with ELISA. (TIF) [file pone.0090613.s001.tif]

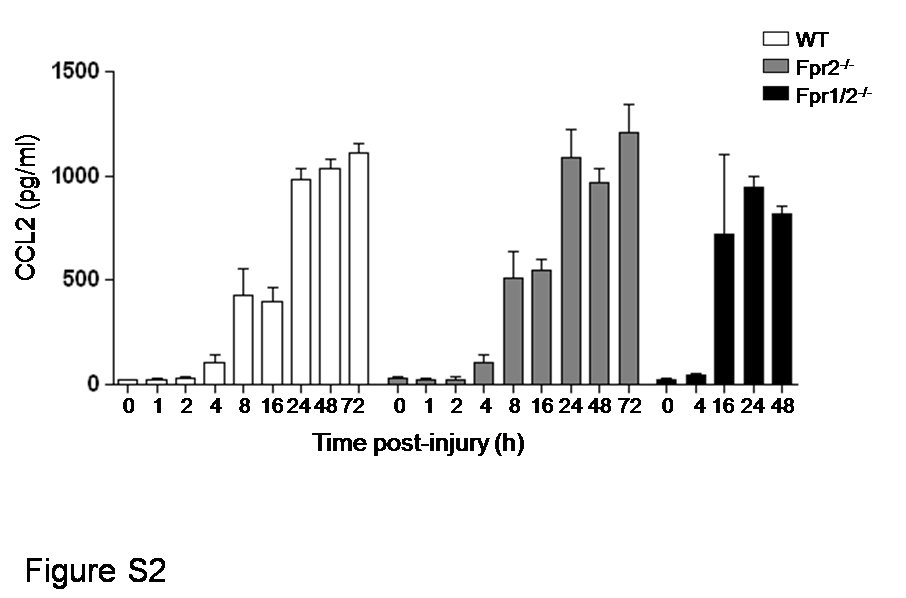

Supplement: Figure S2 — CCL2 production in skin wound. WT and Fpr-deficient mice were subjected to full-thickness skin wound and the wounded skin areas were harvested and homogenized for CCL2 measurement with ELISA. (TIF) [file pone.0090613.s002.tif]
